# Antidepressants in Children and Adolescents: Meta-Review of Efficacy, Tolerability and Suicidality in Acute Treatment

**DOI:** 10.3389/fpsyt.2020.00717

**Published:** 2020-09-02

**Authors:** Katharine Boaden, Anneka Tomlinson, Samuele Cortese, Andrea Cipriani

**Affiliations:** ^1^ Oxford Health NHS Foundation Trust, Warneford Hospital, Oxford, United Kingdom; ^2^ Department of Psychiatry, University of Oxford, Oxford, United Kingdom; ^3^ Center for Innovation in Mental Health, Faculty of Environmental and Life Sciences and Clinical and Experimental Sciences (CNS and Psychiatry), Faculty of Medicine, School of Psychology, University of Southampton, Southampton, United Kingdom; ^4^ Solent NHS Trust, Southampton, United Kingdom; ^5^ Division of Psychiatry and Applied Psychology, School of Medicine and National Institute for Health Research MindTech Mental Health MedTech Cooperative and Centre for ADHD and Neurodevelopmental Disorders Across the Lifespan, Institute of Mental Health, University of Nottingham, Nottingham, United Kingdom; ^6^ New York University Child Study Center, New York, NY, United States

**Keywords:** antidepressants, children and adolescents, systematic review, meta-analysis, efficacy, tolerability, suicidality

## Abstract

Antidepressants are prescribed for the treatment of a number of psychiatric disorders in children and adolescents, however there is still controversy about whether they should be used in this population. This meta-review aimed to assess the effects of antidepressants for the acute treatment of attention-deficit/hyperactivity disorder (ADHD), anxiety disorders (ADs), autistic spectrum disorder (ASD), enuresis, major depressive disorder (MDD), obsessive-compulsive disorder (OCD), and posttraumatic stress disorder (PTSD) in children and adolescents. Efficacy was measured as response to treatment (either as mean overall change in symptoms or as a dichotomous outcome) and tolerability was measured as the proportion of patients discontinuing treatment due to adverse events. Suicidality was measured as suicidal ideation, behavior (including suicide attempts) and completed suicide. PubMed, EMBASE, and Web of Science were systematically searched (until 31 October 2019) for existing systematic reviews and/or meta-analyses of double-blind randomized controlled trials. The quality of the included reviews was appraised using AMSTAR-2. Our meta-review included nine systematic reviews/meta-analyses (2 on ADHD; 1 on AD; 2 on ASD; 1 on enuresis; 1 on MDD, 1 on OCD and 1 on PTSD). In terms of efficacy this review found that, compared to placebo: fluoxetine was more efficacious in the treatment of MDD, fluvoxamine and paroxetine were better in the treatment of AD; fluoxetine and sertraline were more efficacious in the treatment of OCD; bupropion and desipramine improved clinician and teacher-rated ADHD symptoms; clomipramine and tianeptine were superior on some of the core symptoms of ASD; and no antidepressant was more efficacious for PTSD and enuresis. With regard to tolerability: imipramine, venlafaxine, and duloxetine were less well tolerated in MDD; no differences were found for any of the antidepressants in the treatment of anxiety disorders (ADs), ADHD, and PTSD; tianeptine and citalopram, but not clomipramine, were less well tolerated in children and adolescents with ASD. For suicidal behavior/ideation, venlafaxine (in MDD) and paroxetine (in AD) were associated with a significantly increased risk; by contrast, sertraline (in AD) was associated with a reduced risk. The majority of included systematic reviews/meta-analyses were rated as being of high or moderate in quality by the AMSTAR-2 critical appraisal tool (one and five, respectively). One included study was of low quality and two were of critically low quality. Compared to placebo, selected antidepressants can be efficacious in the acute treatment of some common psychiatric disorders, although statistically significant differences do not always translate into clinically significant results. Little information was available about tolerability of antidepressants in RCTs of OCD and in the treatment of ADHD, ASD, MDD, and PTSD. There is a paucity of data on suicidal ideation/behavior, but paroxetine may increase the risk of suicidality in the treatment of AD and venlafaxine for MDD. Findings from this review must be considered in light of potential limitations, such as the lack of comparative information about many antidepressants, the short-term outcomes and the quality of the available evidence.

## Introduction

There are many classes of antidepressants, which include selective serotonin reuptake inhibitors (SSRIs), serotonin-noradrenaline reuptake inhibitors (SNRIs), noradrenaline and specific serotonergic antidepressants (NASSAs), tricyclic antidepressants (TCAs), and monoamine oxidase inhibitors (MAOIs). Antidepressants are one of the possible intervention strategies for a number of mental health conditions in adults as well as in young people. Indeed, several antidepressants are currently licensed for the treatment of child and adolescent psychiatric disorders, with specific indications varying across countries. For instance, in the USA, fluoxetine and escitalopram are Food and Drug Administration (FDA)-approved for major depressive disorder (MDD), fluoxetine, sertraline, fluvoxamine, and clomipramine for obsessive-compulsive disorder (OCD), duloxetine for generalized anxiety disorder (GAD) and the combination of olanzapine and fluoxetine for bipolar depression (1). In the UK, fluoxetine is licensed for MDD, fluvoxamine and sertraline for OCD, and imipramine for nocturnal enuresis (2). Furthermore, some antidepressants are used by clinicians for non-licensed indications, such as amitriptyline for neuropathic pain and, historically, TCAs, in particular imipramine, have been used for the management of attention-deficit/hyperactivity disorder (ADHD).

Over the past decade, the use of antidepressants in children and adolescents has increased in many Western countries. From 2005 to 2012, the prevalence of antidepressant use has increased from 1.3% to 1.6% in the USA, from 0.7% to 1.1% in the UK, from 0.6% to 1.0% in Denmark, from 0.5% to 0.6% in Netherlands, and from 0.3% to 0.5% in Germany (1).

Despite this increase in the rate of prescriptions and notwithstanding their licensed indication for a number of disorders, the use of antidepressants in children and adolescents remains controversial. In particular, the efficacy and tolerability of antidepressants for MDD in young people have been questioned, in the light of a high placebo response rate ranging from 22% to 62% (2) and the “black box warning” issued by the FDA in 2004, advising of the increased risk of suicidal behaviors among children treated with SSRIs (3). The FDA warning was based on an analysis published more than one decade ago about industry-sponsored randomized controlled trials (RCTs). However, since then, an increasing number of studies have questioned the methodological rigor of the FDA analysis (4).

To shed light on this clinically relevant question, a comprehensive and rigorous evidence synthesis may support the discussion and the clinical decision-making among patients, prescribers and policy makers. To the best of our knowledge, no meta-review has been published to comprehensively summarize the findings of all available secondary studies in the field.

## Method

We conducted a meta-review of the existing literature on RCTs in children and adolescents across a number of disorders (see list below). We followed the Preferred Reporting Items for Systematic Reviews and Meta-Analyses (PRISMA) approach (5). The protocol of this meta-review is available online (https://www.psych.ox.ac.uk/team/andrea-cipriani).

We searched PubMed, EMBASE, and Web of Science from database inception to 31^st^ October 2019 for systematic reviews and meta-analyses of double-blind RCTs on the use of orally-administered antidepressants in the treatment of children and adolescents (aged 18 or below) with a diagnosis of anxiety disorder (AD), ADHD, autistic spectrum disorder (ASD), Enuresis, MDD, OCD, and posttraumatic stress disorder (PTSD) according to standard operationalized criteria such as Diagnostic and Statistical Manual of Mental Disorders (DSM)-III, DSM-III-R, DSM-IV(TR), DSM-5, International Classification of Diseases (ICD)-10 or Research Diagnostic Criteria. We searched a long list of antidepressants [based on Cipriani et al. (6)], including amitriptyline, bupropion, citalopram, clomipramine, desipramine, duloxetine, escitalopram, fluoxetine, fluvoxamine, imipramine, mirtazapine, milnacipran, nefazodone, nortriptyline, paroxetine, sertraline, tianeptine, and venlafaxine. Our complete search strategy is detailed in the protocol and in the **Supplementary Table 1**. We manually searched the reference lists of selected publications for additional relevant articles.

The titles and abstracts of all references were screened for eligibility by three authors (KB, AT, SC). Data extraction was performed by KB and double-checked by AT or SC. Full-texts of potentially eligible references were then retrieved and assessed for inclusion. Disagreement in the selection of pertinent papers was resolved with discussion (also involving the fourth author, AC).

We included systematic reviews/meta-analyses in English language. Reviews including trials recruiting participants with comorbid physical health conditions or psychiatric disorders (e.g., substance abuse, psychosis, etc.) were excluded, as were any reviews involving combination therapy. Reviews focusing on treatment resistant depression or relapse prevention were also excluded. In light of concerns raised regarding the potential underreporting of negative findings from RCTs of SSRI use in childhood depression (7), reviews were also excluded if they did not search for or include unpublished trial data (this is a clarification and small deviation from the original protocol, where we said that “reviews will be excluded if they do not include data from unpublished trials”).

Where more than one systematic review/meta-analyses on the treatment of a diagnosis of interest was identified, the most recent and comprehensive was selected. If the most recent review was not the most comprehensive, preference was given to the most comprehensive.

In order to ensure all relevant RCTs were considered in this meta-review, the list of studies included in the most comprehensive systematic review/meta-analysis was cross-checked against the list in all other identified systematic reviews/meta-analyses on the same diagnosis. If an RCT relevant to the present meta-review was not included in the retained systematic review/meta-analysis, it was manually retrieved and reviewed against our inclusion criteria.

Relevant information was extracted from the included systematic reviews, including aim(s), intervention(s), population, methodology, outcomes and their evaluation: (i) efficacy; (ii) tolerability; and (ii) suicidality. Efficacy was measured as response to treatment (either as mean overall change in symptoms or as a dichotomous outcome) and tolerability was measured as the proportion of patients discontinuing treatment due to adverse events. Suicidality was measured as suicidal ideation, behavior (including suicide attempts) and completed suicide.

The quality of the retrieved systematic reviews was assessed using AMSTAR-2 (A MeaSurement Tool to Assess Systematic Reviews), a practical critical appraisal tool to enable health professionals and policy makers to carry out rapid and reproducible assessments of the quality of conduct of systematic reviews of RCTs of interventions (8).

## Results

**Figure 1:**
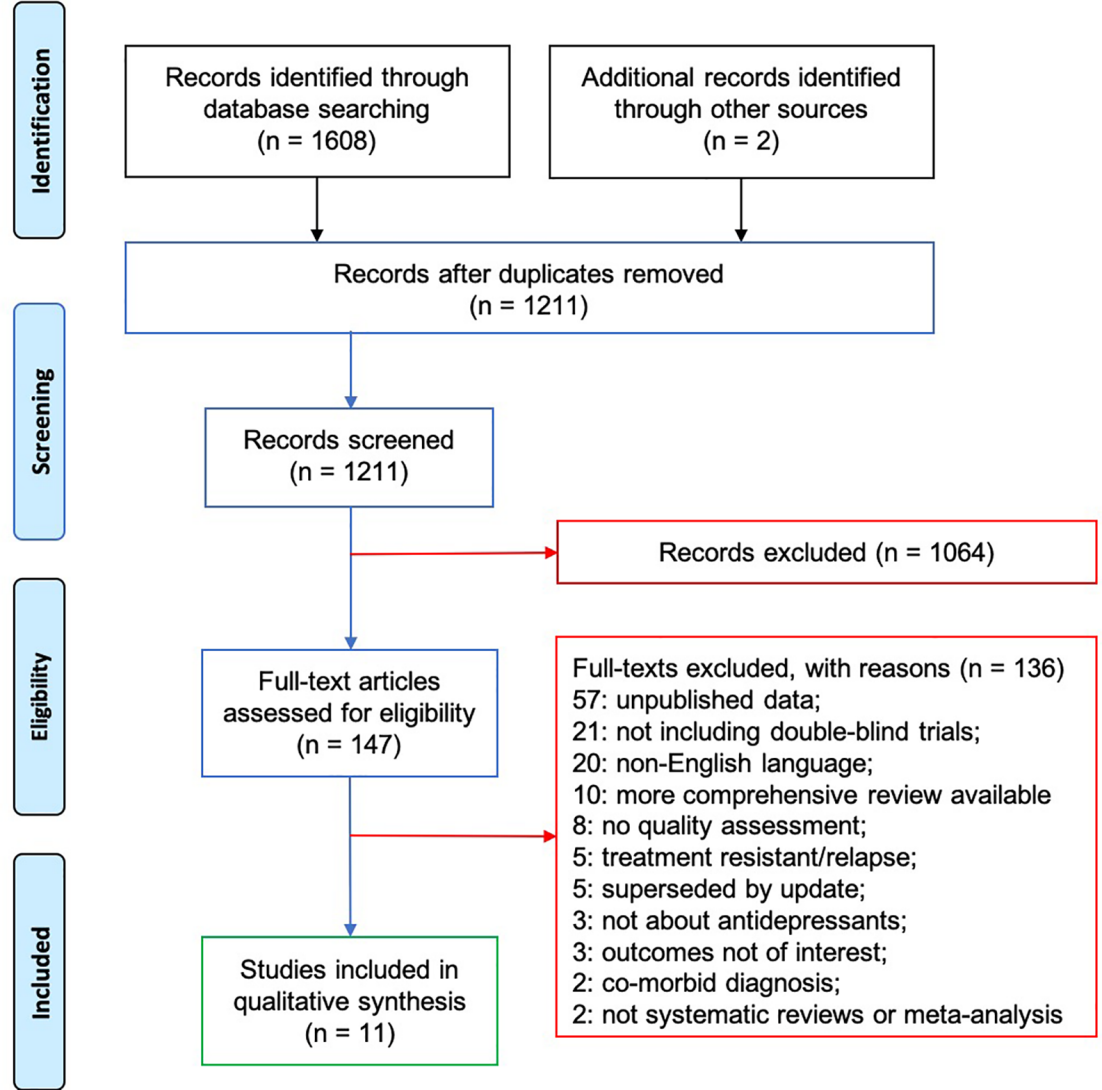
PRISMA Diagram.

The search returned 1,211 unique references and we retrieved the full text of 147 studies. Eleven references were initially considered as relevant to the research question (**Figure 1**). Of the systematic reviews/meta-analyses identified, two concerned the use of antidepressants for treatment of ADHD (9, 10); one for ADs (11); two focused on ASD, one on SSRIs (12) and one on TCAs (13); one on enuresis (14); one on MDD (6), one on OCD (15) and one on PTSD (16). In addition, cross-referencing with the other eligible systematic reviews/meta-analyses identified two individual RCTs in Tsapakis et al. (17) that were not included in Cipriani et al. (6) [Avci et al. (18); Simeon et al. (19)].

The characteristics and outcomes of each of the included systematic reviews/meta-analyses are presented in **Table 1**. Based on AMSTAR-2, overall only one review (network meta-analysis) was rated as high quality (10), five reviews were considered moderate in quality (6, 9, 12, 13, 15), one low quality (16), and two critically low quality (11, 14) (see **Table 2** and **Supplementary Table 2**).

**Table 1:** Description and results of individual systematic reviews and meta-analyses included in the meta-review.

Study (year)	Population	Drug		Primary outcomes	Results (versus placebo)
**ADHD**					
**Efficacy**					
Cortese et al. (10)	40 children (6–17) meeting DSM-IV-TR criteria for ADHD	Bupropion		Change in severity of ADHD core symptoms based on clinicians’ ratings (completed with parents) as measured by ADHD-RS-IV	SMD −0.96; 95% CI −1.69 to−0.22*
				Change in severity of ADHD core symptoms based on teachers’ ratings as measured by ADHD-RS-IV	SMD −0.32; 95% CI −1.07 to 0.43*
Otasowie et al. (9)	91 children and adolescents (6–17 years) with diagnosis of ADHD (DSM-III criteria)	Desipramine		Reduction in ADHD symptoms based on teachers’ ratings as measured by the Conners Teacher Questionnaire and CABRS	SMD −0.97; 95% CI −1.66 to −0.28
**Tolerability**					
Cortese et al. (10)	40 children (6–17) meeting DSM-IV-TR criteria for ADHD	Bupropion		Proportion of participants who left the study because of any side-effect	OR 1.51; 95% CI 0.17 to 13.27
**ANXIETY DISORDERS**		
**Efficacy**					
Dobson et al. (11)	1,847 children and adolescents (6–17 years) diagnosed with mixed anxiety disorders, generalized anxiety disorder (GAD), social anxiety disorder, school phobia, separation anxiety disorder, elective mutism	Fluvoxamine		Treatment response as measured by CGI-I scores ≤ 3	Log OR 2.1; 95% CrI 0.3 to 3.9
				Improvement in anxiety symptom severity as measured by PARS	MD 8.3; 95% CrI 2.5 to 14.3
		Fluoxetine		Treatment response as measured by CGI-I scores ≤ 2	Log OR 1.4; 95% CrI 0.4 to 2.9
				Improvement in anxiety symptom severity as measured by PARS, SPAI-C and MASC	MD 2.6; 95% CrI −1.4 to 8.2
		Paroxetine		Treatment response as measured by CGI-I scores ≤ 2	Log OR 1.3; 95% CrI −0.4 to 3.0
				Improvement in anxiety symptom severity as measured by LSAS-CA	MD 18.4; 95% CrI 4.1 to 32.4
		Sertraline		Treatment response as measured by CGI-I scores ≤ 2	Log OR 1.8; 95% CrI 0.8 to 3.9
				Improvement in anxiety symptom severity as measured by PARS, HARS	MD 3.0; 95% CrI −0.1 to 9.6
		Duloxetine		Treatment response as measured by 50% improvement on PARS	Log OR 0.7; 95% −1.0 to 2.4
				Improvement in anxiety symptom severity as measured by PARS	MD 2.8; 95% CrI −2.8 to 8.5
		Venlafaxine		Treatment response as measured by CGI-I scores ≤ 2	Log OR 0.9; 95% CrI −0.1 to 1.9
				Improvement in anxiety symptom severity as measured by PARS	MD 2.6; 95% CrI −1.1 to 5.9
		Clomipramine		Treatment response as measured by CGI-I scores ≤ 2	Log OR 0.2; 95% CrI −1.5 to 1.9
				Improvement in anxiety symptom severity as measured by MASC	MD -5.9; 95% CrI −19.0 to 7.7
		Imipramine		Treatment response as measured by CGI-I scores ≤ 2 and Global Improvement	Log OR 1.1; 95% CrI −0.5 to 2.7
				Improvement in anxiety symptom severity (measure not reported)	MD 3.1; 95% CrI −4.5 to 10.7
**Tolerability**					
Dobson et al. (11)	1,847 children and adolescents (6–17 years) diagnosed with mixed anxiety disorders, generalized anxiety disorder (GAD), social anxiety disorder, school phobia, separation anxiety disorder, elective mutism	Fluvoxamine		Early discontinuation due to adverse event	Log OR −2.1; 95% CrI −7.0 to 2.4
		Fluoxetine			Log OR −2.5; 95% CrI −7.9 to 1.8
		Paroxetine			Log OR −1.7; 95% CrI −6.0 to 2.5
		Sertraline			Log OR −1.7l 95% CrI −6.6 to 2.8
		Duloxetine			Log OR −0.2; 95% CrI −4.3 to 3.9
		Venlafaxine			Log OR 0.8; 95% CrI −2.1 to 3.8
		Clomipramine			Log OR −1.5; 95% CrI −6.8 to 3.3
		Imipramine			Log OR −16.6; 95% CrI −83.7 to 37.5
**Suicidality**					
Dobson et al. (11)	1,847 children and adolescents (6–17 years) diagnosed with mixed anxiety disorders, generalized anxiety disorder (GAD), social anxiety disorder, school phobia, separation anxiety disorder, elective mutism	Fluvoxamine		Treatment-emergent suicidality	No data
		Fluoxetine			No data
		Paroxetine			Log OR −20.0; 95% CrI −60.4 to −1.7
		Sertraline			Log OR 19.8; 95% CrI 0.7 to 61.7
		Duloxetine			Log OR −0.2; 95% CrI −2.8 to 2.5
		Venlafaxine			Log OR −1.4; 95% CrI −5.2 to 1.4
		Clomipramine			No data
		Imipramine			Log OR −17.3; 95% CrI −54.8 to 0.1
**ASD**					
**Efficacy**					
Hurwitz et al. (13)	42 children and adolescents with a diagnosis of an autism spectrum disorder (DSM-IIIR and Autism Diagnostic Interview criteria; ICD-10	Tianeptine		Inappropriate speech as measured by parents’ and teachers’ ratings using the Aberrant Behavior Checklist	placebo 6.1 (SD = 2.5), tianeptine 4.2 (SD = 3.8); p = 0.042
		Tianeptine		Inadequate eye contact as measured by parents’ and teachers’ ratings using the Aberrant Behavior Checklist	placebo 8.2 (SD = 5.4), tianeptine 7.4 (SD = 3.6); p = 0.041
		Clomipramine		Reduction in abnormal behaviors as measured by the Autism-relevant subscale of the CPRS	placebo 47 (SD = 8), clomipramine 36 (SD = 8); F = 24.2; df 3,33; p = 0.0001
		Clomipramine		Inappropriate speech as measured by clinicians, based on parental reports and direct observations, using the Autism-specific subscale of the CPRS	placebo 4 (SD = 2), clomipramine 3 (SD = 2); F = 1.4, df = 3,33; p = 0.27
Williams et al. (12)	193 children and adolescents (3–17 years) with a diagnosis of an autism spectrum disorder (ADOS, ADIR, DISCO, CARS or diagnostic criteria as defined by DSM-IV or ICD-10, that is Pervasive Developmental Disorder, excluding Rett Syndrome and Childhood Disintegrative Disorder).	Fluoxetine		Changes in CGI scale adapted to Global Autism	No statistical data provided
		Citalopram		Changes in parents’ ratings using the six subscales of the RBS-R	p > 0.36
**Tolerability**					
Hurwitz et al. (13)	42 children and adolescents with a diagnosis of an autism spectrum disorder (as above)	Tianeptine		Levels of drowsiness as measured by symptom checklist designed by study group	placebo 1.4 (SD = 2.3), tianeptine 2.9 (SD= 2.4); p = 0.022
		Tianeptine		Levels of activity as measured by symptom checklist designed by study group	placebo 2.4 (SD = 3.3), tianeptine 4.0 (SD = 3.7); p = 0.029
		Clomipramine		No. of adverse events as measured by the Subjective Treatment Emergent Symptoms Subscale	No statistical data provided
Williams et al. (12)	193 children and adolescents (3–17 yeas) with a diagnosis of an autism spectrum disorder (as above)	Fluoxetine		Frequency of adverse effects as measured by a side effects symptom checklist	No statistical data provided
		Citalopram		One or more treatment-emergent adverse events as measured by Safety Monitoring Uniform Report Form	placebo 86.8%, citalopram 97.3%; p = 0.03
**MAJOR DEPRESSIVE DISORDER**					
**Efficacy**					
Cipriani et al. (6)	5260 children and adolescents (9–18 years) with a primary diagnosis of major depressive disorder according to standardised diagnostic criteria.	Fluoxetine		Mean overall change in depressive symptoms as measured by the CDRS-R, HAMD, BDI, CDI	SMD −0.51; 95% CI −0.99 to −0.03
		Desipramine			SMD −0.45; 95% CI –1.52 to 0.62
		Duloxetine			SMD −0.35; 95% CI –1.24 to 0.54
		Venlafaxine			SMD −0.26; 95% CI –1.10 to 0.58
		Mirtazapine			SMD −0.24; 95% CI –1.25 to 0.77
		Sertraline			SMD −0.23; 95% CI –1.21to 0.77
		Citalopram			SMD −0.18; 95% CI –1.18 to 0.82
		Escitalopram			SMD −0.17; 95% CI –1.15 to 0.81
		Paroxetine			SMD −0.16; 95% CI −0.86 to 0.54
		Nefazodone			SMD −0.15; 95% CI –1.14 to 0.85
		Imipramine			SMD −0.01; 95% CI −0.98 to 0.95
		Amitriptyline			SMD 0.08; 95% CI –1.45 to 1.61
		Clomipramine			SMD −0.32; 95% CI –1.90 to 1.25
		Nortriptyline			SMD –1.14; 95% CI -2.02 to −0.25
**Tolerability**					
Cipriani et al. (6)	5260 children and adolescents (9–18 years) with a primary diagnosis of major depressive disorder according to standardised diagnostic criteria.	Fluoxetine		Proportion of patients who discontinued treatment due to any adverse event	OR 1.03; 95% CI 0.50 to 2.70
		Desipramine			OR 2.85; 95% CI 0.83 to 21.80
		Duloxetine			OR 2.8; 95% CI 1.20 to 9.42
		Venlafaxine			OR 3.19; 95% CI 1.01 to 18.70
		Mirtazapine			OR 1.36; 95% CI 0.41 to 10.99
		Sertraline			OR 2.94; 95% CI 0.94 to 17.19
		Citalopram			OR 1.13; 95% CI 0.45 to 3.66
		Escitalopram			OR 1.64; 95% CI 0.46 to 13.49
		Paroxetine			OR 1.59; 95% CI 0.77 to 3.95
		Nefazodone			OR 1.29; 95% CI 0.30 to 21.89
		Imipramine			OR 5.49; 95% CI 1.96 to 20.86
		Amitriptyline			OR 0.10; 95% CI 0.02 to 32.16
		Clomipramine			OR 0.79; 95% CI 0.12 to 2.75
		Nortriptyline			OR –1.14; 95% CI -2.02 to −0.25
**Suicidality**					
Cipriani et al. (6)	5260 children and adolescents (9–18 years) with a primary diagnosis of major depressive disorder according to standardised diagnostic criteria.	Fluoxetine		Rates of suicidal behavior or ideation	OR 0.90; 95% CI 0.49 to 1.49
		Desipramine			No data
		Duloxetine			OR 1.07; 95% CI 0.51 to 2.0
		Venlafaxine			OR 0.13; 95% CI 0.00 to 0.55
		Mirtazapine			No data
		Sertraline			OR 0.57; 95% CI 0.06 to 2.05
		Citalopram			OR 0.89; 95% CI 0.22 to 2.53
		Escitalopram			OR 1.08; 95% CI 0.33 to 2.57
		Paroxetine			OR 0.89; 95% CI 0.22 to 2.17
		Nefazodone			No data
		Imipramine			OR 0.42; 95% CI 0.09 to 5.35
		Amitriptyline			No data
		Clomipramine			OR 1.41; 95% CI 0.18 to 5.33
		Nortriptyline			No data
**ENURESIS**					
**Efficacy**					
Meadow and Berg (20) in Sureshkumar et al. (14)	27 children and adolescents (5–13 years) with primary diagnosis of daytime urinary incontinence with or without the presence of nocturnal enuresis.	Imipramine		Levels of dryness as measured by no. of completely dry days in a 4-week period and levels of wetness as measured by severity of wetness on a scale of 0 (none) to 3 (severe)	p > 0.05
				Difference in maximum functional bladder capacity	p > 0.05
**OCD**					
**Efficacy**					
Ipser et al. (15)	765 children and adolescents diagnosed with anxiety disorders (DSM-III. DSM-III-R, DSM-IV diagnostic criteria).	Fluoxetine		Treatment response for OCD as measured by CGI-I scores ≤ 2	RR 2.27; 95% CI 1.35 to 3.80
		Fluvoxamine			RR 1.88; 95% CI 0.94 to 3.76
		Paroxetine			RR 1.41; 95% CI 1.00 to 1.98
		Sertraline			RR 1.61; 95% CI 1.07 to 2.43
		Fluoxetine		Reduction in symptom severity for OCD as measured by the CY-BOCS	MD -5.49; 95% CI -8.63 to -2.36
		Fluvoxamine			MD -2.7; 95% CI -5.76 to 0.36
		Paroxetine			MD -3.44; 95% CI -5.65 to –1.23
		Sertraline			MD -3.82; 95% CI -5.83 to –1.81
		Clomipramine			MD -8.9; 95% CI –12.73 to -5.07
**PTSD**					
**Efficacy**					
Robb et al. (21) in Locher et al. (16)	131 children and adolescents (6–17 years) who met DSM-IV criteria for PTSD.	Sertraline		Reduction in symptom severity as measured by end point change in the UCLA PTSD-I score	MD -3.1; 95% CI -7.9 to 1.7 p = 0.20
				Reduction in PTSD symptoms and functional impairment as measured by parent/guardian ratings using the Child Stress Disorders Checklist	Least squares mean change score: placebo –17.3, sertraline –12.4; p = 0.025
				Treatment response as measured by CGI-S scores ≤ 2	Least squares mean change score: placebo - 1.8, sertraline - 1.4; p = 0.031
				Quality of life as measured by change in Paediatric Quality of Life Enjoyment and Satisfaction Questionnaire score	Least squares mean change score: placebo 10.7, sertraline 7.2; p = 0.037

*Indirect comparison derived from the Network (not pairwise) Meta-Analysis. ADHD, Attention Deficit Hyperactivity Disorder; ADHD-RS-IV, Attention Deficit/Hyperactivity Disorder Rating Scale Fourth Version; ADIR, Autism Diagnostic Interview Revised; ADOS, Autism Diagnostic Observation Schedule; AE, Adverse Events; ASD, Autism Spectrum Disorder; BDI, Beck Depression Inventory; CABRS, Conners Abbreviated Rating Scale; CARS, Childhood Autism Rating Scale; CDI, Children’s Depression Inventory; CDRS-R, Children’s Depression Rating Scale – Revised; CGI-I, Clinical Global Impression – Improvement; CI, Confidence Interval; CPRS, Comprehensive Psychopathological Rating Scale; CrI, Credible Interval; CY-BOCS, Children’s Yale-Brown Obsessive Compulsive Scale’ DISCO, Diagnostic Interview for Social and Communication Disorders; DSM, Diagnostic and Statistical Manual; DSM-IV-TR, Diagnostic and Statistical Manual Fourth Version-Text Revision; HAMD, Hamilton Depression Scale; HARS, Hamilton Anxiety Rating Scale; ICD, International Classification of Disease; LSAS-CA, Liebowitz Social Anxiety Scale for Children and Adolescent; MASC, Multidimensional Anxiety Scale for Children; MD, Mean Difference; OCD, Obsessive-Compulsive Disorder; OR, Odds Ratio; PARS, Paediatric Anxiety Rating Scale; PTSD, Posttraumatic Stress Disorder; RBS-R, Repetitive Behavior Scale – Revised; RR, Risk Ratio; SAS-CA, Social Anxiety Scale for Children and Adolescents; SMD, Standard Mean Difference; SPAI-C, Social Phobia and Anxiety Inventory for Children; UCLA PTSD-I, University of California at Los Angeles Post–Traumatic Stress Disorder Index.

**Table 2:** Summary AMSTAR-2 ratings (A MeaSurement Tool to Assess systematic Reviews).

Systematic review/meta-analysis	AMSTAR-2 Rating
**ATTENTION DEFICIT HYPERACTIVITY DISORDER**	
Cortese et al. (10)	High
Otasowie et al. (9)	Moderate
**ANXIETY DISORDERS**	
Dobson et al. (11)	Critically Low
**AUTISM SPECTRUM DISORDER**	
Hurtwitz et al. (13)	Moderate
Williams et al. (12)	Moderate
**ENURESIS**	
Sureshkumar et al. (14)	Critically Low
**MAJOR DEPRESSIVE DISORDERS**	
Cipriani et al. (6)	Moderate
**OBSESSIVE COMPULSIVE DISORDER**	
Ipser et al. (15)	Moderate
**POSTTRAUMATIC STRESS DISORDER**	
Locher et al. (16)	Low

See text and online appendix for details.

### Anxiety Disorders

The NMA by Dobson et al. (11) compared the use of SSRIs, SNRIs and TCAs (imipramine, clomipramine, fluoxetine, fluvoxamine, sertraline, paroxetine, venlafaxine, and duloxetine) versus placebo in the treatment of ADs (including GAD, mixed AD, social AD, separation AD, school phobia, and elective mutism).

Fluvoxamine was found to be superior to placebo in terms of treatment response reported as log OR (2.1, 95% CrI 0.3 to 3.9) and also in terms of improvement in symptom severity measured by the Pediatric Anxiety Rating Scale (mean difference 8.3, 95% CrI 2.5 to 14.3). Interestingly, sertraline, paroxetine, and fluoxetine were more efficacious than placebo, but only according to one and not both outcome measures.

In terms of tolerability, there were no significant differences between any active treatment and placebo; however treatment-emergent suicidality was significantly greater in paroxetine-treated patients compared to those receiving placebo (log OR 19.5, 95% CrI 1.7 to 60.4), sertraline (log OR 43.5, 95% CrI 10.1 to 96.0), and duloxetine (log OR 20.3, 95% CrI 1.5 to 67.7).

### Attention-Deficit/Hyperactivity Disorder

In the network meta-analysis (NMA) by Cortese et al. (10) bupropion (the only antidepressant included in this NMA) was found to be significantly more efficacious compared to placebo on the severity of ADHD symptoms when rated by clinicians (Standardized Mean Difference (SMD) –0·96, Confidence Interval (CI) 95% –1·69 to –0·22) using the Attention Deficit/Hyperactivity Disorder Rating Scale Fourth Version (ADHD-RS-IV), although teacher ratings were not significant (SMD –0·32, CI 95% –1·07 to 0·43) as measured by the ADHD-RS-IV. These results were derived from indirect analyses within the network, rather than pairwise analyses.

Two relevant studies were included in the second systematic review, which focused on the use of the TCA desipramine for ADHD (9). Desipramine was significantly more efficacious than placebo in treating ADHD symptoms as rated by teachers (SMD −0.97; 95% CI −1.66 to −0.28).

No significant difference was found between bupropion and placebo in terms of tolerability (10); for desipramine no serious adverse events were reported (9). No data on suicidality were reported.

### Autism Spectrum Disorder

Of the two systematic reviews investigating the use of antidepressants in the treatment of children and adolescents with ASD, the first was on TCAs and reviewed the effects of clomipramine and tianeptine on core features of the disorder (autistic symptoms, abnormal eye contact, inappropriate speech) (13). Tianeptine was found to have a significant effect on the improvement of inadequate eye contact and inappropriate speech as rated by parents and teachers on the Aberrant behavior Checklist after 12 weeks compared to placebo (p = 0.041 and 0.042, respectively), although these results were not supported by clinician ratings. Clomipramine was found to be more effective than placebo in reducing abnormal behaviors as rated by the Autism-relevant subscale of the CPRS (p = 0.0001). By contrast, for inappropriate speech there were no statistically significant differences between clomipramine and placebo (p = 0.27).

A second systematic review focused on SSRIs (fluoxetine and citalopram) for the treatment of core features of ASD at 12 weeks (12). No significant differences were found between citalopram and placebo or fluoxetine and placebo on any of the rating scales used by the researchers.

For TCAs (13), tianeptine was shown to significantly increase drowsiness (p = 0.022) and decrease activity (p = 0.029). For clomipramine, there was no statistical significance between active treatment and placebo in the reporting of adverse effects. For SSRIs (12), citalopram was less well tolerated than placebo (p = 0.03), but there were no significant differences in the frequency or severity of adverse effects between fluoxetine and placebo.

In Williams et al. (12) no significant differences between fluoxetine and control groups on the suicide subscale of the Overt Aggression Scale (OAS) were reported, but no data were provided.

### Enuresis

We identified an individual RCT [Meadow and Berg (20)] on the use of antidepressants in the treatment of enuresis within a systematic review by Sureshkumar et al. (14) on the treatment of daytime urinary incontinence in children. The study, which focused on imipramine, concluded that active treatment did not significantly increase maximum functional bladder capacity and there was no significant difference between wetness and dryness scores between imipramine and placebo (full results were not provided, only the p value was reported). No data was available on tolerability or suicidality.

### Major Depressive Disorder

The NMA (6) included 34 RCTs investigating 14 antidepressants and placebo. It concluded that, of all included antidepressants (amitriptyline, citalopram, clomipramine, desipramine, duloxetine, escitalopram, fluoxetine, imipramine, mirtazapine, nefazodone, nortriptyline, paroxetine, sertraline, and venlafaxine), only fluoxetine (data taken from 9 RCTs) was more effective than placebo (SMD −0.51, 95% CrI −0.99 to −0.03) in improvement of depressive symptoms, while nortriptyline was significantly less efficacious (1.14, 2.02 to 0.25). In terms of tolerability, imipramine (OR 5.49, 1.96 to 20.86), venlafaxine (3.19, 1.01 to 18.70) and duloxetine (2.80, 1.20 to 9.42) were significantly less well tolerated compared with placebo.

Venlafaxine was associated with a significantly increased risk of suicidal behavior or ideation when compared with placebo (OR 0.13, 95% CrI 0.00 to 0.55) and also with five other active antidepressants (namely, escitalopram, imipramine, duloxetine, fluoxetine, and paroxetine).

### Obsessive-Compulsive Disorder

The systematic review of pharmacotherapy for the treatment of OCD in children and adolescents concluded that, compared with placebo, fluoxetine (RR 2.27, 95% CI 1.35 to 3.80) and sertraline (1.61, 95% CI 1.07 to 2.43) were both significantly more efficacious in terms of CGI-I score (15). In terms of symptom severity reduction as measured by the Children’s Yale-Brown Obsessive Compulsive Scale (CY-BOCS), all active treatments except fluvoxamine (−2.70, 95% CI −5.76 to 0.36) were found to significantly reduce total symptoms, with clomipramine the most efficacious (-8.90, 95% CI −12.73 to −5.07), compared to placebo. In this meta-analysis, no cases of completed suicide were reported for any of the included RCTs (no other information about tolerability of antidepressants in OCD was reported).

### Posttraumatic Stress Disorder

An RCT on the use of sertraline in the treatment of PTSD (21) was included in the systematic review/meta-analysis by Locher et al. (16) and it concluded no difference between sertraline and placebo using the primary outcome measure (University of California at Los Angeles Post–Traumatic Stress Disorder Index). Results rated by secondary outcome measures favoured placebo over sertraline (Child Stress Disorders Checklist (CSDC), −17.3 vs. −12.4; p=0.025; Clinical Global Impression–Severity Scale (CGI-S), −1.8 vs. −1.4; p = 0.031; Paediatric Quality of Life Enjoyment and Satisfaction Questionnaire (PQ-LES-Q), +10.7 vs. +7.2; p = 0.037).

In the same individual study, a similar proportion of patients reported experiencing at least one adverse event on sertraline (76.1%) and placebo (75.8%). An increase in suicidality from baseline was reported by a slightly higher proportion of patients treated with sertraline (11%) vs. placebo (8%). No cases of completed suicide were reported for the RCT on PTSD (16).

## Discussion

In this meta-review assessing the efficacy, tolerability and risk of suicidality of antidepressants across a number of psychiatric disorders in children and adolescents, we found evidence that, compared to placebo: (1) only fluoxetine was more efficacious, with a moderate effect but a large confidence interval, in decreasing the severity of depressive symptoms in acute MDD; (2) fluvoxamine and paroxetine were significantly better in decreasing the severity of symptoms of ADs; (3) fluoxetine and sertraline were significantly more efficacious in terms of treatment response in OCD; (4) bupropion and desipramine were significantly more efficacious in improving clinician and teacher-rated ADHD symptoms, respectively, albeit with large confidence intervals, reflecting the paucity of studies included in the meta-analysis; (5) clomipramine and tianeptine were superior on some of the core symptoms of ASD, even though data were derived from one study only for each of these two medications; (6) none of the antidepressants was more efficacious for PTSD and enuresis.

With regard to tolerability, compared to placebo: (1) imipramine, venlafaxine, and duloxetine were less well tolerated in young people with acute major depression; (2) no significant differences were found for any of the antidepressants in the treatment of ADs, ADHD, and PTSD; (3) tianeptine and citalopram, but not clomipramine, were less well tolerated in children with ASD. No information about tolerability of antidepressants for enuresis, OCD, PTSD was reported, but it is available in other reviews that did not meet our inclusion criteria [see for instance Caldwell et al. (22)].

Finally, in terms of suicidal behavior/ideation venlafaxine in children/adolescents with depression and paroxetine in those with ADs, respectively, were associated with a significantly increased risk, sertraline was associated with a reduced risk in youth with anxiety, and no cases of completed suicides were reported in studies of OCD and PTSD.

Overall, the evidence from our meta-review is only partially in line with the current license status of antidepressants in children and adolescents. In fact, our findings support: (1) the current license of fluoxetine for MDD; (2) the approval of fluoxetine and sertraline for OCD [e.g., USA (1); UK (2)]; (3) the lack of approval of antidepressants for ADHD. However, our results are in contrast with: 1) the absence of license for fluvoxamine and paroxetine for ADs [e.g., US (1), UK (2), France (23)]; 2) the FDA approval of escitalopram for acute and maintenance treatment of depression (24)]; 3) the license for fluvoxamine and clomipramine for OCD (1).

Also, our findings are only partially consistent with recommendations from available guidelines or expert consensus papers. Indeed, our results are in line with:1) the guidelines from the National Institute of Clinical Care and Excellence (25) on the use of fluoxetine for moderate to severe depression; 2) the lack of endorsement for antidepressants to treat ADHD (26), PTSD (27), or core symptoms of ASD (28). However, our findings are at odds with: 1) expert guidance suggesting the use of imipramine for pediatric enuresis (29); 2) the recommendation to use fluoxetine, rather than fluvoxamine, among the SSRIs, for ADs (30); 3) the NICE guidelines recommending the use of imipramine, albeit only when enuresis has proved resistant to all other treatment options (31). We also note that clomipramine is recommended as a treatment for OCD in children and young people who have not responded to, or been unable to tolerate, other treatments, including SSRIs (32). As our meta-review excluded data on treatment-resistant disorders, it is perhaps not surprising that clomipramine was not featured within the included systematic reviews/meta-analyses.

Our findings, which point to the possible efficacy of at least some of the so-called “antidepressants” for the treatment of a number of psychiatric disorders, lend support to the Neuroscience-based Nomenclature (NbN) initiative (33) which proposes to replace the nomenclature of psychotropics, currently focused on the disorder on which there are supposed to be effective, with their neurobiological mechanism(s) of action. We note that a NbN version for psychotropics in children/adolescents is also available (34) and, for instance, fluoxetine should no longer be referred to as an “antidepressant” but as a “reuptake inhibitor of serotonin”.

It is important to note that some factors restrict the interpretation of our findings on the efficacy of antidepressants in children and adolescents. First, the limited availability and quality of supporting evidence, especially the potential limitations of the primary studies that constitute the evidence based for the systematic reviews included in this paper. The risk of bias and the quality of evidence of the individual studies varied and was assessed with different approaches across the included systematic reviews/meta-analyses. Even though randomised controlled trials are at the top of the hierarchy of evidence and have been used over the past 20 years to assess the effect of pharmacological interventions in children and adolescents (35), they may have limited generalizability and may be prone to sponsorship bias (36). Second, the overall quality of the systematic reviews retained in our meta-review, rated *via* the AMSTAR-2 tool (8), was variable, ranging from *high* for the network meta-analysis on ADHD (10) to *critically low* for the evidence synthesis on anxiety (11) and enuresis (14). In particular, one aspect that varied across the retained systematic reviews/meta-analyses was the analysis of unpublished data. Unpublished data were included in some meta-analyses (e.g., Cipriani et al., (6) on MDD) but not others (e.g., Dobson et al. (11) on AD). This is highly relevant as the selective publication of RCTs and data from RCTs, leading to inaccurate estimates of antidepressants, has been documented (37).

It should also be noted that a high placebo response may impact the estimated efficacy of antidepressants (38). Indeed, a higher placebo response in children and adolescents, compared to adults, with MDD has been reported, alongside a less strong placebo response to the same antidepressants in children and adolescents with Ads (11). In the absence of RCTs including a no-treatment arm, this differential response may be due to differences in the probability of spontaneous recovery in childhood depressive and ADs, respectively (11).

In terms of tolerability, the evidence we gathered showed that, whilst some antidepressants (namely, imipramine, venlafaxine, and duloxetine) were less well tolerated than placebo in the treatment of acute major depression, their tolerability was not statistically different from that of placebo in the treatment of ADs, ADHD, and PTSD. These findings could suggest a less good tolerability of antidepressants in young people with depression as compared to other disorders. However, this conclusion should be taken very cautiously, as the inclusion criteria for the participants, and hence their clinical characteristics that may impact on tolerability, varied across the included meta-analyses.

Our meta-review also focused on the risk of suicidal behavior and ideation, a very relevant and controversial topic (39). Whilst it is reassuring that no significant risk of completed suicide emerged across the retained meta-analyses, we did find that at least some antidepressants, notably venlafaxine in children/adolescents with depression and paroxetine in those with ADs, were associated with a significantly increased risk of suicidal ideation/behaviors. However, we also found that sertraline was associated with a reduced risk in young people with anxiety. This raises the question as to whether the influential but controversial “black box warning” issued by many agencies, including the FDA and European Medicine Agency, stating that antidepressants may increase the risk of suicidal behavior and thinking should be applied to all or to a selected number of antidepressants.

In addition to the methodological issues in accurately estimating the prevalence and magnitude of the problem due to the fact that many trials (especially the older ones) only relied on spontaneous reports of suicidal behavior and/or ideation, we highlight here that, since the warning, there has been a decline in the prescription of antidepressants in young people, alongside an increase in the rate of suicidal events among patients with severe depression (4). These findings have been criticized by other researchers, which reported data from case-control studies that showed increased risk of suicide attempts and suicide among youth taking antidepressants, even after controlling for some relevant confounders (40). For clinical practice, one consistent finding from these reviews is that prediction of suicide is difficult and associated with uncertainty. It is important that this is acknowledged by clinicians and services, and discussed openly with patients, parents and carers. Whether prediction models and risk assessment tools can be applied to suicide prevention remains an open question. Future work needs to move towards real-world clinical evaluations that examine the incremental benefits of using these tools to support clinical decision-making (41).

In addition to the strengths of this meta-review, which was based on a comprehensive search of the literature and an assessment of the quality of the systematic reviews/meta-analyses, a number of limitations should be noted. First, we did not include all available systematic reviews and meta-analyses; rather, we retained only the most comprehensive/recent, so we may have missed some relevant data. It is worth noting that the latest guidance from the Cochrane handbook does not indicate a specific way to select systematic reviews/meta-analyses to be included in meta-reviews; rather, it offers a number of options, including our approach (42).

It is also possible that the retained meta-analyses did not include RCTs that would provide relevant information for the present meta-review, owing to the specific inclusion criteria of each meta-analysis. However, checking the references of the included systematic reviews/meta-analyses, we found only two small studies (60 patients in total) that would not change the results materially (see Results section for details about these trials). We also restricted the search to limited number of antidepressants (see Method section for our protocol) and to articles in English only. It is likely that other pharmacological interventions are also used for the disorders under investigation in this article, however we decided to focus on the antidepressants which are more frequently prescribed in real world practice (43).

Second, the systematic reviews/meta-analyses were focused on RCTs only, which have well-known limitations in terms of duration (the length of the trials included in our meta-review ranged from two to 16 weeks), reporting of adverse events and selection of participants, which limits their external validity in terms of efficacy and tolerability. Even if we searched for unpublished data, it is known that the published literature is also biased substantially as a result of selective outcome reporting (44), not only in terms of overestimated efficacy but also underestimated effects of serious adverse events and harms, such as suicidal ideation and behavior (45).

Results from our analysis should be taken with caution and our meta-review is not informative on the long-term effects of antidepressants in children. Whilst conducting RCTs in the long-term is challenging from a practical and ethical standpoint, the use of discontinuation trials (46), which are still limited in the field, should be encouraged as they can provide evidence on the possible long-term persistence of effects. Also, we did not include information from observational studies that are more suitable to provide data on outcomes not routinely included in RCTs. However, while the challenge of the lack of randomization in observational trials has been, at least in part, addressed by the use of the so called within-individual design studies in some fields, e.g., ADHD (47), the half-life of antidepressants makes the interpretation of within-individual design studies challenging. Moreover, studies retained in our meta-review excluded specific clinical populations, such as treatment patients resistant to previous antidepressants. Finally, it was beyond the scope of our meta-review to provide evidence on how to sequence pharmacological and nonpharmacological treatment.

Third, our meta-review does not allow for the comparison of the efficacy and tolerability of antidepressants across a number of disorders. Therefore, based on our findings it would be misleading to conclude that, for instance, venlafaxine is associated with increased risk of suicidal behavior/ideation in major depression but not in AD, as data for major depression and anxiety, respectively, were derived from two different network meta-analyses with different inclusion/exclusion criteria.

Fourth, as per review protocol, we limited the analysis of adverse events to those that resulted in discontinuation. The number of people dropping out from treatment because of side effects can be considered a pragmatic measure of severity of symptoms, however, this information contributes only to part of the full clinical picture. Adverse events reduce quality of life and therefore may reduce the benefits of antidepressants, despite not resulting in discontinuation. Moreover, specific adverse events—no matter how severe they are—are important for patients and for the shared decision-making process (48). Tolerability, withdrawal effects and dependence on antidepressants is also topical in the current scientific debate, as highlighted by the recent report published on September 2019 by Public Health England (49). We aim to cover this issue in future evidence synthesis projects.

In conclusion, the results from our analysis of aggregate data should be contextualized, incorporating patient’s values and preferences (50). Treatment decisions should be tailored to patients on an individual basis, so we recommend clinicians, patients and policy makers to refer to the evidence provided in the present meta-review and make decisions about the use of antidepressants in children and adolescents taking into account a number of clinical and personal variables (51). The available evidence base is not enough and randomised data should probably not be the only source of information. One way forward is to use comparative analysis of individual patient data in combination with high-quality real-world data to identify effect modifiers and prognostic factors that can inform tailored treatments and shared clinical decision making across a number of psychiatric conditions and interventions (52). This will be a material move towards a real precision-psychiatry approach that may improve the clinical outcome (and quality of life) of our patients (53).

## Data Availability Statement

The datasets generated for this study are available on request to the corresponding author.

## Author Contributions

Protocol was designed by KB, AT, SC, and AC. Search was performed by KB and reference screening undertaken by KB, AT, and SC. Data extraction was performed by KB and double-checked by AT or SC. The manuscript was written by KB, AT, and SC and reviewed by AC. All authors contributed to the article and approved the submitted version.

## Funding

AC is supported by the National Institute for Health Research (NIHR) Oxford cognitive health Clinical Research Facility, by an NIHR Research Professorship (grant RP-2017-08-ST2-006), by the NIHR Oxford and Thames Valley Applied Research Collaboration and by the NIHR Oxford Health Biomedical Research Centre (grant BRC-1215-20005). The views expressed are those of the authors and not necessarily those of the UK National Health Service, the NIHR, or the UK Department of Health.

## Conflict of Interest

Two authors (AC and SC) are authors on two of the systematic reviews included in our meta-review [Cipriani et al. (6); Cortese et al. (10)]. SC declares reimbursement for travel/accommodation expenses and honoraria in relation to lectures/courses delivered for the Association for Child and Adolescent Health (ACAMH), Canadian ADHD Alliance Resource (CADDRA), British Association of Psychopharmacology (BAP), and Healthcare Convention. AC has received research and consultancy fees from INCiPiT (Italian Network for Paediatric Trials), CARIPLO Foundation and Angelini Pharma; he has also organised a workshop about digital mental health sponsored by Angelini Pharma.

The remaining authors declare that the research was conducted in the absence of any commercial or financial relationships that could be construed as a potential conflict of interest.
