# Supplementary material for: Antidepressants in Children and Adolescents: Meta-Review of Efficacy, Tolerability and Suicidality in Acute Treatment
Source: Front Psychiatry. 2020 Sep 2;11:717. doi: 10.3389/fpsyt.2020.00717 (PMC7493620; doi:10.3389/fpsyt.2020.00717)
Supplement: Supplementary file 1 [file DataSheet_1.docx]

**Title:** Meta-review of the efficacy and safety of antidepressants in children and adolescents: An evidence-based approach to inform clinical practice.

Authors: Katharine Boaden, Anneka Tomlinson, Samuele Cortese and Andrea Cipriani

Review Questions:

- What is the evidence base for the efficacy and tolerability of antidepressants for the treatment of Attention Deficit Hyperactivity Disorder (ADHD), Anxiety Disorder (AD), Autistic Spectrum Disorder (ASD), Enuresis, Major Depressive Disorder (MDD), Obsessive Compulsive Disorder (OCD) and Post-Traumatic Stress Disorder (PTSD) in children and adolescents?
- Is there an evidence base for increased suicidality and/or risk of suicide in children and adolescents using antidepressants?

Searches:

We will search Medline, Embase and Web of Science using keywords and MeSH headings related to antidepressants for a range of diagnoses. The following limits will be applied: Human; English Language

| 1 | exp Depressive Disorder/ or depressive disorder.mp.  depression.mp. or exp Depression/  depressive.mp.  dysthymic disorder.mp. or exp Dysthymic Disorder/  dysthmia*.mp.  depression*.mp.  exp Anxiety/ or Anxiety.mp.  Obsessive-Compulsive Disorder.mp. or exp Obsessive-Compulsive Disorder/  obsessive compulsive disorder.mp.  obsessive-compulsive.mp.  ocd.mp.  phobic.mp.  anxiety disorders.mp. or exp Anxiety Disorders/  panic.mp. or exp Panic/  stress disorder*.mp.  phobia*.mp.  exp Stress Disorders, Post-Traumatic/ or post traumatic stress.mp.  post traumatic symptom*.mp.  posttraumatic symptom*.mp.  ptsd.mp.  exp Attention Deficit Disorder with Hyperactivity/  adhd.mp.  attention deficit.mp.  attention deficit hyperactivity.mp.  hyperkinetic syndrome.mp.  autistic disorder.mp. or exp Autistic Disorder/  autis*.mp.  autistic spectrum disorder*.mp.  exp Asperger Syndrome/ or asperger*.mp.  autism spectrum disorder*.mp.  exp Autism Spectrum Disorder/  autistic spectrum condition*.mp.  autism spectrum condition*.mp.  ASD.mp.  exp Enuresis/ or enuresis.mp.  nocturnal enuresis.mp. or exp Nocturnal Enuresis/  monosymptomatic enuresis.mp.  night enuresis.mp.  sleep enuresis.mp.  sleep enuresis.mp.  bed wet*.mp.  monosymptomatic primary nocturnal enuresis.mp.  primary nocturnal enuresis.mp. |
| --- | --- |
| 2 | serotonin uptake inhibitors.mp. or exp Serotonin Uptake Inhibitors/  serotonin uptake inhibitors*.mp.  selective serotonin reuptake inhibitor.mp.  SSRI.mp.  citalopram.mp. or exp Citalopram/  fluoxetine.mp. or exp Fluoxetine/  paroxetine.mp. or exp Paroxetine/  sertraline.mp. or exp Sertraline/  escitalopram.mp.  fluvoxamine.mp. or Fluvoxamine/  serotonin norepinephrine reuptake inhibitor*.mp.  SNRI.mp.  exp "Serotonin and Noradrenaline Reuptake Inhibitors"/  milnacipran.mp. or exp Milnacipran/  venlafaxine.mp. or exp Venlafaxine Hydrochloride/  duloxetine.mp. or exp Duloxetine Hydrochloride/  reboxetine.mp. or exp Reboxetine/  exp Bupropion/ or bupropion.mp.  (noradrenergic and specific serotonergic antidepressants).mp.  NaSSA.mp.  mirtazapine.mp. or exp Mirtazapine/  TCA.mp. or exp Antidepressive Agents, Tricyclic/  tricyclic.mp.  amersergide.mp.  amineptine.mp.  amitriptyline.mp. or exp Amitriptyline/  amoxapine.mp. or exp Amoxapine/  butriptyline.mp.  clomipramine.mp. or exp Clomipramine/  demexiptiline.mp.  desipramine.mp. or exp Desipramine  dothiepin.mp. or exp Dothiepin/  doxepin.mp. or exp Doxepin/  imipramine.mp. or exp Imipramine/  lofepramine.mp. or exp Lofepramine  melitracen.mp.  metapramine.mp.  nortriptyline.mp. or exp Nortriptyline/  noxiptiline.mp.  opipramol.mp. or exp Opipramol/  protriptyline.mp. or exp Protriptyline  quinupramine.mp.  tianeptine.mp.  trimipramine.mp. or exp Trimipramine/ |
| 3 | child.mp. or exp Child/  exp Pediatrics/ or pediatrics.mp.  paediatrics.mp.  pediatric.mp.  paediatric.mp.  child*.mp.  exp Adolescent/ or adolescen*.mp.  toddler*.mp.  teen*.mp.  boy.mp.  boys.mp.  girl.mp.  girls.mp.  puber*.mp.  pubescen*.mp.  prepubescent.mp.  prepuberty*.mp.  schoolchild*.mp.  school age*.mp.  preschool*.mp.  kindergarten.mp.  primary school*.mp.  secondary school*.mp.  elementary school*.mp.  high school*.mp.  highschool*.mp.  youth*.mp.  young person.mp.  young people.mp.  exp Adolescent/ |
| 4 | systematic review*.mp.  meta analy*.mp.  meta review*.mp.  metareview*.mp.  umbrella review*.mp. |

Methods:

*Types of studies:*

This meta-review will include systematic reviews and meta-analyses of double-blind, randomized controlled trials (RCTs) comparing orally-administered antidepressants among themselves or with placebo. We will search Embase, Web of Science and MEDLINE/PubMed from date of database inception to 31^st^ October 2019.

Where multiple systematic reviews and meta-analyses for the conditions being studied exist, the most comprehensive/recent review will be retained.

*Conditions or domains being studied:*

This meta-review will include systematic reviews that focus on the use of antidepressants to treat either: Attention Deficit Hyperactivity Disorder (ADHD), Anxiety Disorder (AD), Autistic Spectrum Disorder (ASD), Enuresis, Major Depressive Disorder (DD), Obsessive Compulsive Disorder (OCD) or Post-Traumatic Stress Disorder (PTSD).

*Participants/population:*

Systematic reviews and meta-analyses of male and female children and adolescents (18 and under) with a primary diagnosis of either Attention Deficit Hyperactivity Disorder (ADHD), Anxiety Disorder (AD), Autistic Spectrum Disorder (ASD), Enuresis, Major Depressive Disorder (DD), Obsessive Compulsive Disorder (OCD) or Post-Traumatic Stress Disorder (PTSD) according to standard operationalised criteria as per DSM-III, DSM-III-R, DSM-IV(TR), DSM-5 and ICD-10 receiving antidepressant medication will be included.

*Inclusion criteria:*

Systematic reviews and meta analyses will be included if they report on human studies of individuals with either Attention Deficit Hyperactivity Disorder (ADHD), Anxiety Disorder (AD), Autistic Spectrum Disorder (ASD), Enuresis, Major Depressive Disorder (DD), Obsessive Compulsive Disorder (OCD) or Post-Traumatic Stress Disorder (PTSD) receiving orally-administered antidepressant medication. Reviews of studies of children and adolescents aged 18 or under in English language only will be included.

*Exclusion criteria:*

In light of concerns raised regarding the potential underreporting of negative findings from RCTs of SSRI use in childhood depression^[[1]](#footnote-1)^, reviews will be excluded if they do not include data from unpublished trials. Reviews will also be excluded if they omit to include an appraisal of the quality of included RCTs.

Reviews that include trials focusing on physical health disorders or substance use disorders as co-morbidities will be excluded, as will any reviews involving combination therapy (i.e., combination of antidepressants, combination of antidepressants with psychotherapy, or other non-psychotherapeutic interventions).

Reviews focusing on treatment resistant depression or relapse prevention, or trials within a review that contain less than 10 participants, will also be excluded.

*Types of interventions:*

This meta-review will examine the evidence base for: (i) efficacy and tolerability; (ii) suicidality and risk of suicide.

*Comparator/control:*

RCTs comparing antidepressants among themselves or with placebo will be included. Studies comparing antidepressants to non-pharmacological interventions or combinations of anti-depressants with psychotherapy, or other non-psychotherapeutic interventions, will be excluded.

*Types of outcome measures:*

In the meta-review, the efficacy of antidepressants will be measured as the response to treatment (either as mean overall change in symptoms or as dichotomous outcome) and tolerability will be measured by proportion of patients discontinuing treatment due to adverse events.

*Data extraction:*

Three reviewers (KB, AT, SC) will independently screen the title and abstract of retrieved references for inclusion. We will then obtain the full text of potentially eligible studies and the two reviewers will independently assess them for inclusion. Reasons for a paper’s exclusion from the review will be documented. Any disagreements will be resolved by a fourth reviewer (AC) provided they have no conflict of interest for the paper in question. Initial disagreement in the selection of pertinent papers will be resolved with discussion by the four authors (KB, AT, SC, AC). Data extraction will follow the structure outlined in the PRISMA 2009 Flow Diagram.

We will use a data collection form to extract the review criteria and characteristics.

Data to be extracted will include publication details (type of review, journal, date), participants (number, inclusion and exclusion criteria, demographic data), details of the outcomes of interest.

*Assessment of study quality:*

The quality of the retrieved systematic reviews and meta-analyses will be assessed using A Measurement Tool to Assess [Systematic Reviews](https://www.sciencedirect.com/topics/psychology/systematic-review) (AMSTAR-2)^[[2]](#footnote-2)^.

*Strategy for data synthesis:*

A narrative synthesis is planned and will be guided by our three review questions. Where multiple systematic reviews and meta-analyses for the conditions being studied exist, the most comprehensive review will be included in the narrative synthesis.

We will also present reviews meeting inclusion criteria in a table.

**Contact details for further information:**

Katharine Boaden

Katharine.boaden@oxfordhealth.nhs.uk

**Organisational affiliation of the review:**

Department of Psychiatry, University of Oxford

**Review team members and organisational affiliations**

Dr Anneka Tomlinson, Department of Psychiatry, University of Oxford

Professor Samuele Cortese, University of Southampton, UK

Professor Andrea Cipriani, Department of Psychiatry, University of Oxford

**Anticipated start date:**

November 2019

**Anticipated completion date:**

January 2020

1. CJ Whittington, T Kendall, P Fonagy, *et al.* **Selective serotonin reuptake inhibitors in childhood depression: systematic review of published versus unpublished data.**

   [Lancet,](https://www.ncbi.nlm.nih.gov/pubmed/15110490) 363 (2004) p. 1341-5. [↑](#footnote-ref-1)
2. B.J. Shea, B.C. Reeves, G. Wells, *et al.* **AMSTAR 2: a critical appraisal tool for systematic reviews that include randomised or non-randomised studies of healthcare interventions, or both**

   BMJ, 358 (2017), p. j4008 [↑](#footnote-ref-2)
