# Supplementary material for: Antidepressants in Children and Adolescents: Meta-Review of Efficacy, Tolerability and Suicidality in Acute Treatment
Source: Front Psychiatry. 2020 Sep 2;11:717. doi: 10.3389/fpsyt.2020.00717 (PMC7493620; doi:10.3389/fpsyt.2020.00717)
Supplement: Supplementary file 2 [file Table_1.docx]

**Supplementary Table 1: Example of Search Strategy (MEDLINE)**

| 1 | exp Depressive Disorder/ or depressive disorder.mp.  depression.mp. or exp Depression/  depressive.mp.  dysthymic disorder.mp. or exp Dysthymic Disorder/  dysthmia*.mp.  depression*.mp.  exp Anxiety/ or Anxiety.mp.  Obsessive-Compulsive Disorder.mp. or exp Obsessive-Compulsive Disorder/  obsessive compulsive disorder.mp.  obsessive-compulsive.mp.  ocd.mp.  phobic.mp.  anxiety disorders.mp. or exp Anxiety Disorders/  panic.mp. or exp Panic/  stress disorder*.mp.  phobia*.mp.  exp Stress Disorders, Post-Traumatic/ or post traumatic stress.mp.  post traumatic symptom*.mp.  posttraumatic symptom*.mp.  ptsd.mp.  exp Attention Deficit Disorder with Hyperactivity/  adhd.mp.  attention deficit.mp.  attention deficit hyperactivity.mp.  hyperkinetic syndrome.mp.  autistic disorder.mp. or exp Autistic Disorder/  autis*.mp.  autistic spectrum disorder*.mp.  exp Asperger Syndrome/ or asperger*.mp.  autism spectrum disorder*.mp.  exp Autism Spectrum Disorder/  autistic spectrum condition*.mp.  autism spectrum condition*.mp.  ASD.mp.  exp Enuresis/ or enuresis.mp.  nocturnal enuresis.mp. or exp Nocturnal Enuresis/  monosymptomatic enuresis.mp.  night enuresis.mp.  sleep enuresis.mp.  sleep enuresis.mp.  bed wet*.mp.  monosymptomatic primary nocturnal enuresis.mp.  primary nocturnal enuresis.mp. |
| --- | --- |
| 2 | serotonin uptake inhibitors.mp. or exp Serotonin Uptake Inhibitors/  serotonin uptake inhibitors*.mp.  selective serotonin reuptake inhibitor.mp.  SSRI.mp.  citalopram.mp. or exp Citalopram/  fluoxetine.mp. or exp Fluoxetine/  paroxetine.mp. or exp Paroxetine/  sertraline.mp. or exp Sertraline/  escitalopram.mp.  fluvoxamine.mp. or Fluvoxamine/  serotonin norepinephrine reuptake inhibitor*.mp.  SNRI.mp.  exp "Serotonin and Noradrenaline Reuptake Inhibitors"/  milnacipran.mp. or exp Milnacipran/  venlafaxine.mp. or exp Venlafaxine Hydrochloride/  duloxetine.mp. or exp Duloxetine Hydrochloride/  reboxetine.mp. or exp Reboxetine/  exp Bupropion/ or bupropion.mp.  (noradrenergic and specific serotonergic antidepressants).mp.  NaSSA.mp.  mirtazapine.mp. or exp Mirtazapine/  TCA.mp. or exp Antidepressive Agents, Tricyclic/  tricyclic.mp.  amersergide.mp.  amineptine.mp.  amitriptyline.mp. or exp Amitriptyline/  amoxapine.mp. or exp Amoxapine/  butriptyline.mp.  clomipramine.mp. or exp Clomipramine/  demexiptiline.mp.  desipramine.mp. or exp Desipramine  dothiepin.mp. or exp Dothiepin/  doxepin.mp. or exp Doxepin/  imipramine.mp. or exp Imipramine/  lofepramine.mp. or exp Lofepramine  melitracen.mp.  metapramine.mp.  nortriptyline.mp. or exp Nortriptyline/  noxiptiline.mp.  opipramol.mp. or exp Opipramol/  protriptyline.mp. or exp Protriptyline  quinupramine.mp.  tianeptine.mp.  trimipramine.mp. or exp Trimipramine/ |
| 3 | child.mp. or exp Child/  exp Pediatrics/ or pediatrics.mp.  paediatrics.mp.  pediatric.mp.  paediatric.mp.  child*.mp.  exp Adolescent/ or adolescen*.mp.  toddler*.mp.  teen*.mp.  boy.mp.  boys.mp.  girl.mp.  girls.mp.  puber*.mp.  pubescen*.mp.  prepubescent.mp.  prepuberty*.mp.  schoolchild*.mp.  school age*.mp.  preschool*.mp.  kindergarten.mp.  primary school*.mp.  secondary school*.mp.  elementary school*.mp.  high school*.mp.  highschool*.mp.  youth*.mp.  young person.mp.  young people.mp.  exp Adolescent/ |
| 4 | systematic review*.mp.  meta analy*.mp.  meta review*.mp.  metareview*.mp.  umbrella review*.mp. |
